# Report of the Diseases of Edinburgh for March, 1809

**Published:** 1809-05

**Authors:** John Roberton


### *Report of the Diseases of Edinburgh for March,* 1809.
By John Roberton, M. D.

The weather, about the beginning of this month, was pleasant during the day; but after sun-set we were enveloped in fogs, probably owing, in some measure, to the easterly winds which then prevailed. One or two nights were so excessively foggy, that the clothes of those who were exposed for a few minutes had deposited upon them a quantity of water, almost similar to what would have happened had it actually rained. This state of the weather was followed by heavy gales of wind, continuing occasionally till near the middle of the month, when it became cold and cloudy. About the beginning of the last third third of the month, the weather still remaining cloudy, we had some showers of rain, which fell occasionally for three or four days; while the easterly winds, still prevailing, rendered the weather both cold and unpleasant. This was succeeded by showers of hail, accompanied and followed by falls of snow; and the weather, till the end of the month, continued excessively cold. The snow during the last days of the month was pretty general, as all the neighbouring hills were covered with it. The equinoctial gales have not been so tremendous as we often experience them.

The barometer, during the greater part of the month, and even during the fogs, stood high; but after this it fell considerably, and began to rise again about the end of the month. The thermometer, till near the end of the month, stood in general from 35 to 40 deg. when it began to fall.

Inflammatory complaints are now much less frequent, and less severe, than they have been for some time past. The consequences of this state of the system, however, seem rather to be gaining ground on us. On a former occasion, I took notice of large tumours appearing on various parts of the body in those who had been affected with severe inflammatory complaints; and I have lately observed this in those in whom, apparently, no inflammatory state of the system existed, to such a degree at least as to excite alarm.

There certainly is, in practice, too little attention paid to the state of the system previous to the appearance of many diseases which we are daily in the habit of seeing; and by thus partially considering the circumstances attending diseases, our success in practice is certainly much more limited than it would be, were we to make our previous investigations more extensively and more minutely than the mere observation of the moment. It is in this way alone that medical men can possibly practice their profession on the most scientific principles, and, consequently, with the greatest success; while those, on the contrary, who are guided solely by the present appearances, however scientifically they may reason, and even practice, in these complaints, can never hope to be so successful as when their views are more extensively directed. There can be no doubt that such states of inflammation, as we frequently witness in this place, are productive of various diseases whose existence is seldom, if indeed ever, attributed to such a cause; and, thus overlooking the the principal, I would almost say the only rule, by the proper use of which we can expect to be successful in removing them, we need not wonder that the success we happen to have is more accidental, consequently much less frequent than what occurs as the result of reasoning and reflection.

By these morbidly accelerated states of the circulation, diseases of a very different description are produced in different individuals, according to the existing state of the body at the time, viz. abscess, dropsy of various kinds, hydrocephalus, &c.; and I have no doubt that phthisis pulmonalis, and many other diseases of the most serious nature, are produced by the same cause.

Typhus, cynanche tonsillaris, catarrh both acute and chronic, and several other chronic complaints, have been the most prevalent.

The fever, as formerly mentioned, has been of a very bad kind, and in some instances, notwithstanding the utmost attention that could be paid, terminated fatally. I have frequently urged the propriety of adopting some plan, not only for the removal, but for the prevention of these widely destroying diseases. Nor has this task been peculiar to myself; the celebrated Dr. Currie's remarks on the result of the adoption of similar measures, cannot be too often stated. He justly and beautifully says, "This is a prospect in which the philanthropist might indulge with more safety, if he could calculate with equal confidence on the *wisdom* as on the *power* of his species." This remark, I regret to say, may with too much truth and justice be applied to every department of human science.

Cynanche tonsillaris still continues very prevalent, and in almost every degree of severity; and catarrh is more general than it was last month. These diseases have been rendered more severe by many of the inhabitants imprudently throwing off, during the few good days in the beginning of the month, some of their additional winter apparel. Blisters to the throat, with gargles and cathartic medicines, remove the first of these affections, and cathartics in general remove the other. In both of them, however, it is absolutely necessary to avoid the use of all stimulating substances, and to preserve the greatest equality of the atmosphere in which the patients are placed.

The chronic disease most prevalent is catarrh, either as a relic of acute catarrh, or such as annually harrasses patients advanced in life during the winter and spring months. The medicines for its removal are of a different kind from those employed in acute catarrh. I alluded to them and to their effects in my former Reports. They are principally blisters applied to the breast, with the inhalation of the steams of vinegar and hot water, and the diligent use of pills composed of g. myrrh, g. ammoniac, squills, and a small proportion of opium. Perseverance in the use of these medicines, even in the worst of cases, always alleviates these complaints, and in many of them effects a cure.

Chronic rheumatism and asthma, particularly among the old and debilitated, are frequently to be met with.—These chronic diseases, notwithstanding all that has been said and written about them, are the most difficult of cure of any that come under the observation of medical men. This circumstance alone ought not to appal, but to excite us to the most vigorous perseverance in investigating the powers of those medicines which may procure relief to that immense mass of individuals who perpetually labour under them. Thus we should have the double comfort of at once relieving distress, and of obtaining those honours to which successful industry, in the attempt to alleviate distress, is entitled.

